# Depletion of the thiol oxidoreductase ERp57 in tumor cells inhibits proliferation and increases sensitivity to ionizing radiation and chemotherapeutics

**DOI:** 10.18632/oncotarget.5746

**Published:** 2015-10-21

**Authors:** Melanie Hussmann, Kirsten Janke, Philip Kranz, Fabian Neumann, Evgenija Mersch, Melanie Baumann, Kirsten Goepelt, Ulf Brockmeier, Eric Metzen

**Affiliations:** ^1^ Institut für Physiologie, Universität Duisburg-Essen, HufelandstraΔe 55, D45122 Essen, Germany

**Keywords:** endoplasmic reticulum, ER chaperone, unfolded protein response, p53, apoptosis

## Abstract

Rapidly growing tumor cells must synthesize proteins at a high rate and therefore depend on an efficient folding and quality control system for nascent secretory proteins in the endoplasmic reticulum (ER). The ER resident thiol oxidoreductase ERp57 plays an important role in disulfide bond formation. Lentiviral, doxycycline-inducible ERp57 knockdown was combined with irradiation and treatment with chemotherapeutic agents. The knockdown of ERp57 significantly enhanced the apoptotic response to anticancer treatment in HCT116 colon cancer cells via a p53-dependent mechanism. Instead of a direct interaction with p53, depletion of ERp57 induced cell death via a selective activation of the PERK branch of the Unfolded Protein Response (UPR). In contrast, apoptosis was reduced in MDA-MB-231 breast cancer cells harboring mutant p53. Nevertheless, we observed a strong reduction of proliferation in response to ERp57 knockdown in both cell lines regardless of the p53 status. Depletion of ERp57 reduced the phosphorylation activity of the mTOR-complex1 (mTORC1) as demonstrated by reduction of p70S6K phosphorylation. Our data demonstrate that ERp57 is a promising target for anticancer therapy due to synergistic p53-dependent induction of apoptosis and p53-independent inhibition of proliferation.

## INTRODUCTION

One of the major challenges of current cancer treatment is the resistance of tumor cells to ionizing radiation (IR) and chemotherapeutical substances. In an urgent need for new drug targets the endoplasmic reticulum (ER) has become a promising candidate since rapidly growing tumor cells upregulate their protein synthesis and therefore depend on efficient folding of nascent secretory proteins in the ER. A set of molecular chaperones such as BiP, calreticulin (CRT), protein disulfide isomerase (PDI) and GRP94 is mandatory to maintain the critical ER balance [1]. To avoid the accumulation of misfolded proteins, these are bound by molecular chaperones, transferred to the ER-associated degradation pathway (ERAD) and get disposed of by the proteasome [2, 3].

One survival mechanism of cells under stress conditions is the activation of the Unfolded Protein Response (UPR) through one of its three ER-stress sensors IRE1, PERK and ATF6. Aim of the UPR is to re-establish ER homeostasis through reduction of protein synthesis and simultaneous overexpression of ER chaperones [4]. Depending on the severity and duration of ER stress, the UPR can be cytoprotective or induce apoptosis. Since the apoptotic response in cancer cells is often attenuated or even abrogated, this complex ER network becomes an important pathway which supports growth [5, 6]. Activation of the UPR can occur after general and reversible attenuation of protein synthesis via eIF2α phosphorylation which is summarized as the Integrated Stress Response (ISR) [7]. In addition to PERK, the ISR can also be mediated by other kinases upon amino acid starvation, viral infection, iron deficiency and oxidative stress. Activation of the ISR results in the synthesis of UPR target genes through increased eIF2α-independent translation of the transcription factor ATF4. Activation of at least one branch of the UPR has been reported in a number of human tumor samples [8]. Consequently, ER chaperones become an attractive target in cancer cells [9, 10]. For instance, the knockdown of BiP enhanced doxorubicin-induced apoptosis not only in proliferating HEp3 cells, but also in a dormant subgroup which usually shows high resistance to chemotherapeutic treatment [11].

The ER provides a more oxidizing environment than the cytosol favoring formation of disulfide bonds in nascent exoproteins which is crucial for their stability and functionality. More than 20 members of the PDI family introduce disulfide bonds into client proteins and catalyze rearrangement of incorrectly formed disulfide bonds. The most prominent member of the PDI family is PDIA1 which exerts oncogenic and pro-survival functions in different cancer types [12, 13]. Interestingly, novel potent PDI inhibitors are being developed, but their clinical value has yet to be proven [14–16]. The closest homologue of PDI is the thiol oxidoreductase ERp57 (GRp58/1,25D3-MARRS Receptor/PDIA3), best known for its structural role in the assembly of the MHC class I molecule [17]. However, similar to PDI, it affects disulfide bond formation and reformation in the processing of numerous client proteins [18]. Both PDI and ERp57 are composed of four distinct domains a, b, b’ and a’ forming a horseshoe shape where the *a* and *a*’ domains carry the active site CGHC and the *b*’ domains of both enzymes contain the substrate binding site [19]. Of note, despite its C-terminal ER retention signal (QEDL), ERp57 was found in the cytoplasm as an interaction partner of mTOR [20] and STAT3 [21] as well as in the nucleus involved in DNA binding [22, 23] and on the cell surface [24, 25].

Although some of the localization and functional studies of ERp57 are not entirely conclusive, ERp57 turned out to be a much more important player than originally anticipated with a cellular impact beyond the ER. Of particular interest is the report of an siRNA-mediated knockdown of ERp57 that increased cell death in two human cancer cell lines initiated by the synthetic drug fenretinide [26] although the authors did not address the question how downregulation of ERp57 was actually linked to apoptosis. Here, we tested the potential benefit of targeting ERp57 in the human cancer cell lines HCT116 and MDA-MB-231 in combination with irradiation and chemotherapeutic compounds.

## RESULTS

### ERp57 modulates apoptosis in response to anticancer treatment

**Figure 1:**
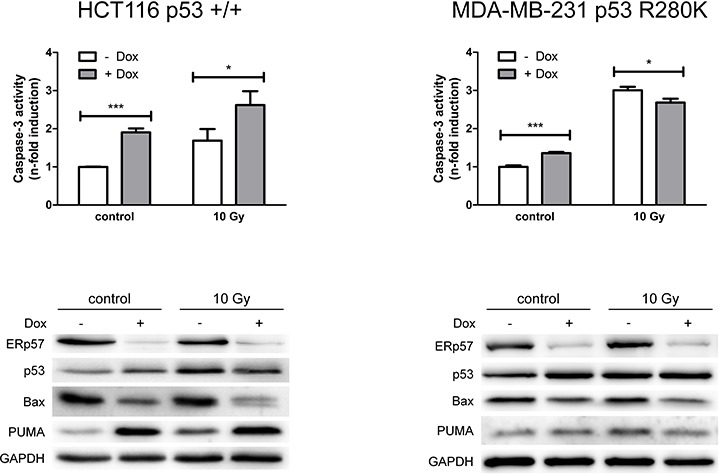
ERp57 modulates irradiation-induced apoptosis in a cell-type specific manner HCT116shERp57 and MDA-MB-231shERp57 were irradiated with 10 Gy 24 h after induction of the ERp57 knockdown. 72 h after irradiation whole cell extracts were tested for caspase-3 activity (upper panel). In parallel, total cell lysates were subjected to Western blotting (lower panel).

**Figure 2:**
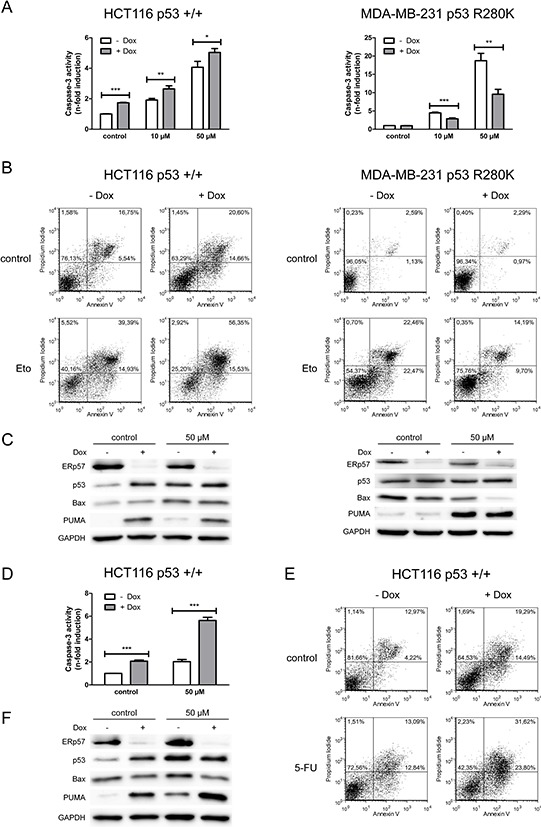
ERp57 modulates chemotherapy-induced apoptosis in a cell-type specific manner For all chemotherapy experiments control cells were treated with vehicle only (DMSO). **A.** Apoptosis was triggered in inducible HCT116shERp57 and MDA-MB-231shERp57 by treatment with etoposide 24 h after induction of the ERp57 knockdown. 48 h after treatment whole cell extracts were tested for caspase-3 activity. **B.** Cells were treated with 50 μM etoposide (Eto) 36 h after induction of ERp57 knockdown. 48 h after treatment apoptosis was quantified by staining with annexin V and PI and subsequent FACS analysis. Representative data of two experiments are shown. **C.** Cells treated as in (B) were subjected to Western blotting. **D.** Cells were treated with 50 μM 5-fluorouracil (5-FU) 24 h after induction of the ERp57 knockdown. 48 h after treatment caspase-3 activity was quantified. **E.** Cells were incubated with 50 μM 5-fluorouracil 36 h after induction of ERp57 knockdown. 48 h after treatment cells were stained with annexin V and PI for FACS. Representative data of two experiments are shown. **F.** In parallel, cell lysates were analysed by Western blotting.

Lentiviral doxycycline-inducible ERp57 knockdown displayed an efficiency of 80–90% on average. Suppression of ERp57 significantly and consistently increased apoptosis in HCT116shERp57 (Fig. 1 and 2A). In contrast, induction of apoptosis following ERp57 knockdown in MDA-MB-231shERp57 cells was observed to vary between no effect and a slight protection against apoptosis (Fig. 1 and 2A).

Following IR the cell lines showed different responses to depletion of ERp57. Wild-type p53 expressing HCT116shERp57 cells displayed enhanced sensitivity to irradiation-induced apoptosis while MDA-MB-231shERp57 cells harbouring mutant p53 (R280K) were protected against irradiation-induced apoptosis after knockdown induction (Fig. 1). In HCT116shERp57 cells p53 was induced by both knockdown of ERp57 and IR whereas the combined treatment did not further increase p53 levels.

**Figure 3:**
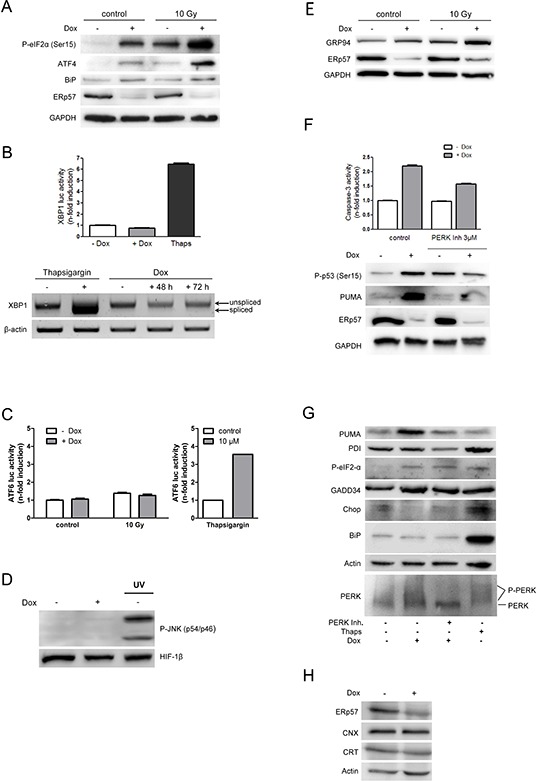
Knockdown of ERp57 in HCT116 cells activates the UPR exclusively via PERK **A.** Inducible HCT116 shERp57 cells were irradiated with 10 Gy 24 h after induction of ERp57 knockdown. 72 h after irradiation the cells were lysed and subjected to Western blotting. P-eIF2α (Ser51), ATF4 and BiP protein levels were monitored as indicators of ER-stress, GAPDH served as a loading control. **B.** XBP1 splicing as an indicator for IRE1 activation was analysed with a luciferase reporter gene assay (upper panel). Cells were transfected with the pXBP1u-FLuc reporter plasmid 24 h after induction of ERp57 knockdown and harvested 48 h later. Firefly luciferase activity was normalized to *renilla* luciferase activity. As a positive control for ER stress, cells were treated with 10 μM thapsigargin for 24 h. Lower panel: total RNA was subjected to RT-PCR and analysed for XBP1 splicing. β-actin served as a loading control. **C.** 24 h after knockdown induction, the cells were transiently transfected with an ATF6-luciferase reporter gene construct. After 48 h lysates were prepared and analysed by luciferase activity detection. **D.** 96 h after induction of ERp57 knockdown, P-JNK was detected by Western blotting as an indicator of IRE1 activation. Hif-1β was used as a loading control and UV-treated cells as a positive control for JNK activation. A representative Western blot from two independent experiments is shown. **E.** Cells were treated as in (A) and tested for GRP94 as an indicator of ERAD, GAPDH served as a loading control. **F.** After ERp57 knockdown induction and treatment with 3 μM PERK inhibitor for 96 h, cell extracts were tested for caspase-3 activity (upper panel). Representative data of two experiments are shown. In parallel, cell lysates were subjected to Western blotting (lower panel). Representative Western blots from two experiments are shown. **G.** Cells were treated as in (F) Cell lysates were analysed by Western blotting. Phosphorylated PERK is detected as a higher molecular weight smear. Western blots from two independent experiments are shown. **H.** After ERp57 knockdown induction for 96 h, cell lysates were analysed by Western blotting. A representative Western blot from two independent experiments is shown.

However, while the pro-apoptotic p53 target Bax was reduced following suppression of ERp57 (Fig. 1), another pro-apoptotic p53 target, PUMA, was strongly induced. In MDA-MB-231shERp57 the induction of p53 and PUMA following ERp57 knockdown was much less pronounced (Fig. 3C). Reduced apoptosis correlated with reduced Bax protein levels in MDA-MB-231 which is in line with the differing effects on apoptosis induction.

Following etoposide treatment similar effects of ERp57 knockdown were observed. In line with a protective effect of ERp57 in HCT116shERp57, these cells showed increased etoposide-induced apoptosis after doxycycline treatment. In contrast, MDA-MB-231shERp57 cells were protected against etoposide-induced apoptosis upon ERp57 knockdown (Fig. 2A). These alterations were also reflected by changes in early and late apoptotic/necrotic fractions of the cells upon double staining with annexin V and propidium iodide (PI) (Fig. 2B). While knockdown of ERp57 increased the apoptotic fraction from 22% to 35% in untreated HCT116shERp57 cells, these changes were not observed in MDA-MB-231shERp57 cells. Treatment with 50 μM etoposide without knockdown induced apoptosis to a similar extent of approximately 50% in both cell lines, whereas suppression of ERp57 induced opposite effects in the two cell lines when combined with etoposide. In HCT116shERp57 cells combined treatment increased the apoptotic fraction from 54% to 72%. In contrast, suppression of ERp57 reduced etoposide-induced apoptosis from 45% to 24% in MDA-MB-231shERp57 cells. Similar to the results observed for IR, apoptosis induction in HCT116shERp57 correlated with the induction of p53 and PUMA while the amount of Bax protein was not altered (Fig. 2C). In MDA-MB-231shERp57 cells ERp57 suppression did not lead to pronounced changes in p53 and PUMA although PUMA was strongly induced following treatment with etoposide. Interestingly, Bax was generally reduced upon ERp57 knockdown in the breast cancer cells which coincided with the reduction of apoptosis particularly upon treatment with etoposide where a reduction of apoptosis and Bax protein was observed. The apoptotic response of HCT116shERp57 cells to 5-fluorouracil was similar to the response to etoposide (Fig. 2D, 2E and 2F).

### Knockdown of ERp57 activates the PERK branch of the UPR selectively

To assess whether the apoptotic response in HCT116 cells is caused by unfolded proteins in the ER, all branches of the UPR were tested following suppression of ERp57 in untreated and irradiated cells. Although an induction of ATF4 and eIF2α phosphorylation at Ser51 was observed which indicates inhibition of protein translation (Fig. 3A), an induction of XBP1reporter gene activity or mRNA splicing (Fig. 3B) or ATF6 (Fig. 3C) reporter gene activity was not detectable. ER-stress has been reported to activate the JNK-pathway via IRE1 [27]. However, we were not able to detect stimulation of the JNK pathway after depletion of ERp57 (Fig. 3D). Together with the activation of ATF4 (Fig. 3A) these results point to a selective activation of the PERK branch of the UPR. In addition, increased expression of the abundant ER chaperone and ERAD component GRP94 (Fig. 3E) was detectable. Upregulation of PDI (Fig. 3G) or calreticulin or calnexin (Fig. 3H) were undetectable and induction of BiP occurred only inconsistently upon ERp57 knockdown (Fig. 3A and 3G). In irradiated cells we detected similar phosphorylation levels of eIF2α and a similar increase in GRP94 as observed after ERp57 suppression. Following ERp57 inhibition and irradiation the more pronounced induction of eIF2α phosphorylation and ATF4 protein expression demonstrated a further increase in ER stress levels (Fig. 3E). However, ATF6 activity and further induction of BiP protein levels were not detectable after knockdown of ERp57 in irradiated cells (Fig. 3A and 3C). Interestingly, GRP94 expression further increased under these conditions (Fig. 3E). To examine a direct involvement of the activated PERK branch in the apoptotic response, we treated the cells with the specific PERK inhibitor GSK2656157 [28]. The inhibitor reduced caspase-3 activity, phospho-p53 and PUMA (Fig. 3F and 3G). Of note, different results were obtained for the two pro-apoptotic factors after ATF4 activation: while CHOP remained low or was even decreased, GADD34 was elevated (Fig. 3G). Taken together these results indicate that depletion of ERp57 does not induce apoptosis via full activation of the UPR but enables selective activation of the PERK branch which triggers the induction of cell death.

### Loss of ERp57 promotes apoptosis in a p53-dependent manner

**Figure 4:**
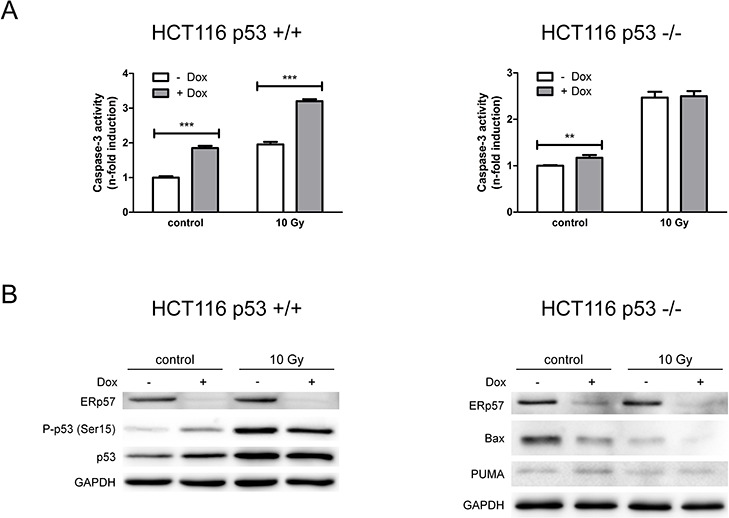
Loss of ERp57 induces p53-dependent apoptosis **A.** Apoptosis was triggered in inducible HCT116 shERp57 p53 +/+ and p53 −/− cells by irradiation with 10 Gy 24 h after induction of ERp57 knockdown. 72 h after irradiation caspase-3 activity was measured. **B.** In parallel, total cell lysates were subjected to Western blotting.

To prove that the induction of apoptosis is indeed dependent on p53, the isogenic HCT116 cell line deficient for p53 was tested for the response to IR in the presence and absence of ERp57. HCT116shERp57 p53 −/− cells showed a less prominent increase in apoptosis after ERp57 knockdown induction as compared to p53 wild-type cells (Fig. 4A). In addition, ERp57-dependent changes were not observed in irradiation-induced apoptosis in p53-deficient cells. Wild-type p53 induced in HCT116shERp57 cells upon treatment with doxycycline was phosphorylated at Ser15 demonstrating targeted activation of the tumor suppressor (Fig. 4B). In addition, the induction of PUMA observed in the p53 +/+ cells was not present upon depletion of ERp57 in p53-deficient cells while Bax protein levels were strongly reduced (Fig. 4B).

**Figure 5:**
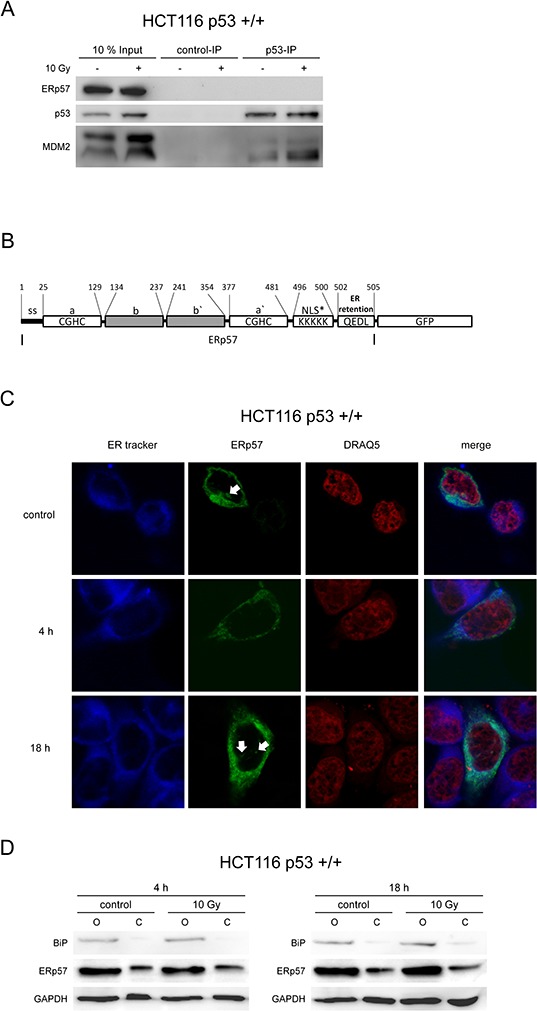
ERp57 and p53 do not interact directly **A.** Whole cell lysates were prepared from HCT116 cells 24 h following treatment with 10 Gy and subjected to immunoprecipitation of p53. ERp57 was not detectable by immunoblotting in contrast to MDM2. As a negative control immunoprecipitation was performed with an isotype control IgG. **B.** Domain structure of the ERp57-GFP fusion protein used for fluorescence localization. NLS*: potential nuclear import signal, SS: signal sequence. **C.** Fluorescence of ERp57-GFP. 24 h following transient transfection with ERp57-GFP (green) HCT116 cells were exposed to 10 Gy and fixed at the indicated time points. ER and nucleus were counterstained with ER-tracker (blue) and DRAQ5 (red), respectively. Arrows point to spurious nuclear signals. **D.** Subcellular fractionation showing the subcellular localization of endogenous ERp57. HCT116 cells were exposed to 10 Gy and subcellular fractionation with digitonin was performed at the indicated time-points to separate the cytoplasm (C) from the organelle fraction (O) BiP was monitored as a marker for the organelle fraction while GAPDH indicates extraction of cytoplasm.

To further investigate whether a direct interaction between p53 and ERp57 is involved in protection against apoptosis, p53 was immunoprecipitated from untreated and irradiated HCT116 cells. ERp57 was undetectable in p53 pulldown samples while the known interaction partner MDM2 co-precipitated with p53 (Fig. 5A).

As ERp57 contains a potential nuclear localization signal (Fig. 5B) and has previously been described to be located not only to ER and cytoplasm but also to the nucleus [22, 29], we tested whether ERp57 is involved in transcriptional regulation. Thus, a GFP-tagged fusion protein was introduced into HCT116 to determine the subcellular localization of ERp57 (Fig. 5B). Counterstaining of ER and nucleus demonstrated that ERp57-GFP is localized in the perinuclear space but not inside the nucleus in HCT116 cells (Fig. 5C). False positive signals in the nucleus were disproven by z-stack confocal microscopy. A fraction of the protein localized to the cytoplasm as demonstrated by permeabilisation of the plasma membrane with digitonin. Importantly, the integrity of ER membranes was not compromised as the ER resident protein BiP was not extracted (Fig. 5D). The extraction of cytoplasmic fluid was proven by detection of GAPDH.

### ERp57 triggers proliferation of cancer cells independent of the p53 status

**Figure 6:**
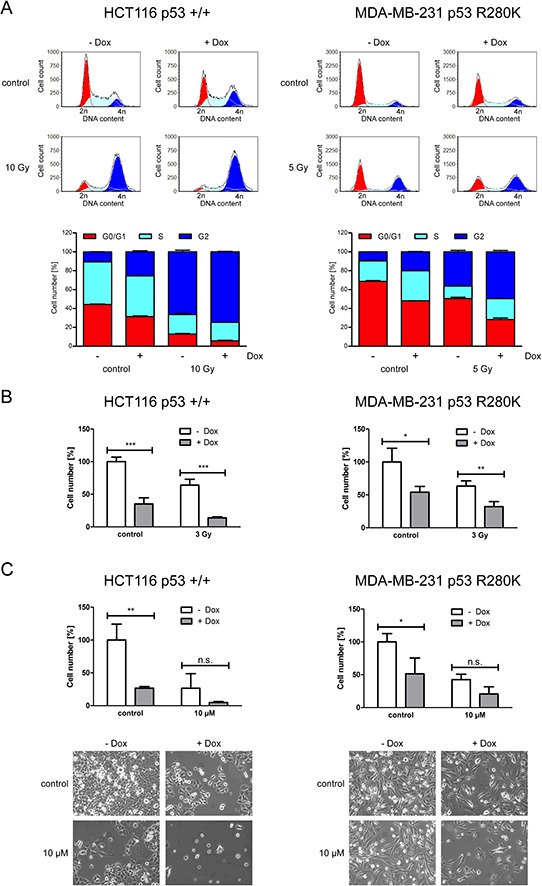
ERp57 triggers proliferation and cell cycle progression of cancer cells independently of p53 **A.** Inducible HCT116 shERp57 and MDA-MB-231 shERp57 were irradiated 48 h after induction of ERp57 knockdown. 24 h after irradiation cell cycle distribution (upper panel) was determined by PI staining and subsequent FACS analysis. In the lower panel fractions of the cells in each cell cycle phase are plotted. Representative data of two independent experiments are shown. **B.** 4d after knockdown induction HCT116 shERp57 and MDA-MB-231 shERp57 cells were irradiated with 3 Gy. Following treatment cells were reseeded. Cell numbers were determined when the control samples had grown to confluency and normalized to non-induced controls. **C.** 5 d after knockdown induction, cells were treated with 10 μM etoposide for 4 h. Cell numbers were determined as in (B) Images of representative samples were taken prior to counting.

Next, we examined the impact of ERp57 on cell cycle progression of HCT116 and MDA-MB-231. In both cell lines knockdown of ERp57 induced cell cycle arrest in the G2-phase while the amount of cells in the G0/G1-phase was reduced (Fig. 6A). For HCT116shERp57 cells the effect of combined treatment with irradiation and knockdown of ERp57 on G2 arrest was more pronounced than for MDA-MB-231 cells. Similar results were obtained for cellular proliferation. For both cell lines cell numbers were significantly reduced upon knockdown of ERp57 (Fig. 6B). Upon incubation with 10 μM etoposide cell numbers were not significantly altered by suppression of ERp57 (Fig. 6C). In summary, as opposed to the distinct apoptotic responses, HCT116shERp57 and MDA-MB-231shERp57 cells showed similar changes in proliferation following knockdown of ERp57.

**Figure 7:**
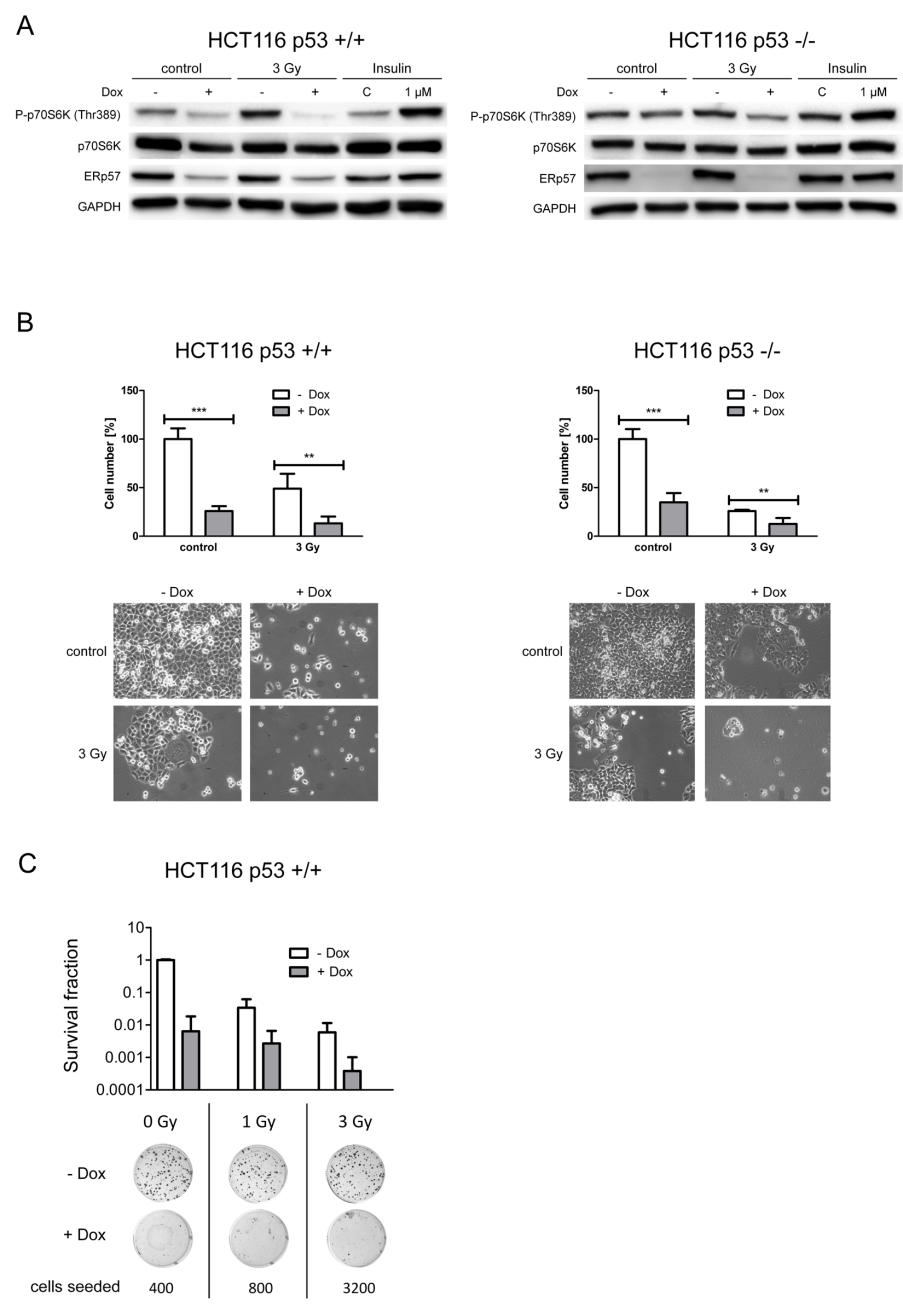
ERp57 triggers proliferation via mTOR activation in a p53-independent manner **A.** HCT116 shERp57 p53 +/+ and p53 −/− cells were exposed to 3 Gy 24 h after induction of ERp57 knockdown. 72 h after irradiation cell lysates were subjected to Western blotting. mTOR activity was monitored via phosphorylation status of p70S6K at Thr389. As a positive control cells were treated with 1 μM insulin for 1 h. **B.** HCT116 shERp57 p53 +/+ and p53 −/− cells were treated with 3 Gy 4 days after knockdown induction. Following treatment cells were reseeded. Cell counts were determined when the control samples were grown to confluency and normalized to non-induced controls. Images of representative samples taken prior to cell number determination are shown for each subfigure (lower panel). **C.** Colony formation assay of HCT116 p53 +/+ cells. After induction of ERp57 knockdown, cells were irradiated with 1 or 3 Gy and incubated for 10–14 days.

To examine p53-dependence of the proliferation response HCT116 p53 proficient cells were compared to the isogenic p53 deficient cells following ERp57 knockdown. ERp57 has been shown to be involved in regulation and assembly of mammalian target of rapamycin complex 1 (mTORC1). As a measure for mTORC1 activity we monitored phosphorylation of p70S6K at Thr389 in p53 proficient and deficient cells following knockdown of ERp57. We observed decreased activity irrespective of the presence of p53, albeit less pronounced in p53 −/− cells (Fig. 7A). This effect was even stronger when cells were treated with IR in addition. In line with these changes proliferation was significantly decreased in HCT116shERp57 cells in the presence and absence of p53 (Fig. 7B). We further performed clonogenic survival assays to test radiation sensitivity of HCT116 p53 +/+ cells after ERp57 depletion (Fig. 7C). ERp57 depletion alone led to a loss of colony formation as effective as treatment with 3 Gy. In combination however, this effect was amplified between 12- and 15-fold depending on the radiation dose.

## DISCUSSION

One metabolic trait which distinguishes most tumor cells from normal cells is that, despite harsh microenvironmental conditions such as hypoxia, nutrient deprivation and low pH, they must synthesize proteins at a high rate and therefore show an upregulation of the ER folding machinery per se which leads to continuous perturbation of the ER and activation of ERAD. When the ER cannot cope with the level of misfolded proteins, the unfolded protein response (UPR) results in reversible inhibition of protein translation, triggers expression of ER chaperones and may in the case of persistent malfunction tip the balance from pro-survival activity to apoptosis. However, a general benefit for tumor patients through UPR-activation remains highly controversial, as most tumor cells as compared to non-cancerous cells bypass apoptotic stress responses during prolonged ER stress and even worse, show increased resistance to chemotherapeutics due to upregulation of ER chaperones, in particular BiP [30–33]. On the other hand, several studies have demonstrated that treatment with ER stressing agents sensitizes tumor cells to alkylating agents like cisplatinum and melphalan [34, 35]. In addition, human A549 lung cancer cells showed ER stress-mediated inhibition of DNA double-strand break repair that enhanced radiosensitivity [36]. Encouraged by previous work [12] which provided evidence that global inhibition of PDI activity mediated by bacitracin can boost the apoptotic effect of chemotherapeutic drugs, our initial hypothesis was that depletion of ERp57 in tumor cells would result in severe ER stress and favour cell death, even more pronounced in combination with IR.

The ER luminal oxidoreductase ERp57 (EC5.3.4.1), also termed GRP58 or PDIA3, is involved in disulfide bond formation of exoproteins. Indeed, ablation of ERp57 triggered apoptosis and magnified the impact of IR and chemotherapeutics in human colon cancer HCT116 cells which was dependent on a crosstalk between the ER and functional p53. The triple negative breast cancer cell line MDA-MB-231 carrying mutant p53, however, did not show enhanced apoptosis, although the knockdown efficiency was comparable between both cell lines. In our hands ERp57 was not detectable inside the nucleus and thus contradicts other studies that suggested a nuclear role for ERp57 in DNA binding, repair and gene activation [22, 37].

In HCT116 cells, we detected a very distinct cellular response via the PERK branch only, whereas neither IRE1 nor ATF6 showed activation after ERp57 knockdown alone. In addition, we did not observe more than basal protein levels of the ER chaperones calnexin, CRT and the ER master sensor BiP. These findings are in line with previous reports since induction of these proteins is primarily driven by activated ATF6 through binding to ER stress response elements (ERSEs) in their promotor regions [38]. In an earlier study, ERp57 was targeted with specific siRNA in the human endothelial cell line EA. hy926 and, in contrast to our results, decreased the apoptotic response. The authors also noticed an upregulation of BiP which potentially explains the cell protective effect [39]. Remarkably, the only ER chaperone that was affected by ERp57 depletion in our study was GRP94 [40]. Interestingly, the substrates of GRP94 are believed to be disulfide-bonded proteins exclusively [41] which are prone to misfolding and aggregation after downregulation of the thiol oxidoreductase ERp57. It seems possible that the exclusive upregulation of GRP94 is a specific response to boost the disposal of misfolded client proteins of ERp57 but prevents a full UPR at the same time. A phenotype similar to ours was obtained in a former study of human hepatocellular carcinoma cells where all tested ER chaperones were unaffected by single knockdown and even by double knockdown of ERp57 with PDI accomplished by siRNA gene silencing [42]. The striking phenotypes found in many studies which used toxic chemical ER stress inducers such as thapsigargin or tunicamycin are unlikely to reflect the much more nuanced outcome of ER stress caused by hypoxia, ROS or nutrient deprivation and may thus be grossly misleading [43]. In support of this, a previous study examined the impact of individually silenced ER chaperones and postulated very distinct cellular stress responses [44]. Hence, it is possible that the so called “classical” UPR with a full activation of all three branches is either a very transient or a quite rare event *in vivo*.

Activation of the PERK- eIF2α pathway results in a global inhibition of cap-dependent protein translation but stimulates translation of the basic leucine-zipper transcription factor ATF4, the master regulator of the ISR. Two of its target genes are GADD34 and CHOP, both are pro-apoptotic factors with short half-lives, but with different action patterns: GADD34 is a cofactor of protein phosphatase 1 (PP1) which dephosphorylates eIFα after prolonged ER stress to initiate apoptosis eventually through an overload of protein translation [45, 46]. CHOP (also known as GADD153) on the other hand is a transcription factor itself and mediates upregulation of the pro-apoptotic factors PUMA, BIM and DR5 after ER stress [47–49]. Remarkably, although we detected PERK-mediated ER stress in HCT116 cells after ERp57 knockdown followed by a strong PUMA expression, we did not observe any CHOP induction at all. This observation bears the question how p53-dependent apoptosis is linked to ER stress in this scenario. In fact, the ablation of ERp57 led to upregulation of GADD34 which was suggested by others to play an additional, more direct role in apoptosis via phosphorylation of cytoplasmic p53 [50, 51]. Presumably, a protein interaction of GADD34 with p53 could also compete for binding to PP1. This might explain why we detected phosphorylated eIF2α despite enhanced GADD34 levels. Moreover, a very recent study described a new aspect of the interplay between disturbed ER homeostasis, cytoplasmic p53 and the Ca^2+^-dependent mitochondrial apoptosis [52]. The authors demonstrate that activated cytoplasmic wild type p53 can bind and modulate SERCA pumps at the ER, followed by [Ca^2+^] transfer from the ER to mitochondria ultimately leading to cell death. Interestingly, this non-nuclear pro-apoptotic effect of p53 is abolished in mutants defective in DNA binding thus emphasizing the crucial role of the DNA binding domain for pro-apoptotic functions of WT p53. Apart from its function as an anti-apoptotic factor in WT p53 expressing tumor cells, our data provided strong evidence that ERp57 acts as an indispensable growth factor, notably independent of the p53 status of the cell. This effect on proliferation is probably carried out by the cytosolic fraction of ERp57, at least partly through stabilizing the mTORC1 complex to promote phosphorylation of its downstream target S6K1 as reported here and by others [20]. In addition we noticed an enhanced G2-M arrest after ERp57 depletion which is particularly interesting since this is the most radiosensitive phase of the cell cycle [53]. At this point we do not know how ERp57 interferes in cell cycle progression. However, an ER stress induced G2 arrest via PERK was described previously, but in contrast to our results the authors showed a strict dependency on p53 [54]. Instead, cytoplasmic ERp57 may function as an activator of phase-specific cyclin-dependent kinases [55]. Whether any of the contributions of ERp57 requires its oxidoreductive activity or is based on structural properties has not been addressed yet. Unfortunately, it is not possible yet to compare the effects of shRNA-mediated knockdown of ERp57 with a specific inhibitor of ERp57. Clearly, further work is needed to unravel the cellular impact of ERp57 on cell cycle and proliferation which is the object of an ongoing investigation in our lab.

In conclusion, our data demonstrate that depletion of ERp57 leads to a significant induction of p53-dependent apoptosis. Although classical ER stress was not detectable at various time points after ERp57 knockdown, we noticed a selective activation of PERK and an upregulation of the ERAD protein GRP94 which may suggest that compensatory effects in certain cell type specific or metabolic situations are possible when ERp57 is downregulated. We also demonstrate that a minor proportion of ERp57 is localized in the cytoplasm where it promotes cell growth and proliferation at least partly via activation of mTOR irrespective of the p53 status. Importantly, p53-dependent and p53-independent effects were synergistic which suggests that the effect of ERp57 inhibition is reduced in a p53-deficient cellular background but not eliminated. Taken together, our study provides compelling evidence that inhibition of ERp57 affects proliferation and apoptosis of cancer cells and that the development of specific inhibitors for ERp57 could help to improve the outcome of future cancer therapies.

## MATERIALS AND METHODS

### Reagents, cell culture, transfection and lentiviral transduction

PERK inhibitor GSK2656157 was from Millipore. All other chemical compounds were from Sigma. MDA-MB-231 and HEK293T cells were cultured in high-glucose DMEM (Invitrogen, Darmstadt, Germany). HCT116 p53+/+ and isogenic p53−/− cell lines (generously provided by B. Vogelstein, Baltimore, MD) were cultured in McCoy's 5A medium (Lonza, Basel, Switzerland). Media were supplemented with 10% FBS and antibiotics. For transient transfection TurboFect (Thermo Scientific) was used. Lentiviral particles were produced in HEK293T cells as described previously [56]. For transduction 2 × 10^5^ cells were incubated with 2 × 10^6^ transduction units for 16–24 h in the presence of 8 μg/μl polybrene. Cells were selected by treatment with the appropriate concentration of puromycin for approximately 10 d. pLKO.1-shRNA-ERp57 tet-on (tetracycline inducible) contained the sequence GGAATAGTCCCATTAGCAAAG of ERp57 mRNA (GenBank acc. no. NM_005313). Gene knockdown was induced by addition of 250 ng/ml doxycycline.

### Apoptosis quantification

Caspase-3 activity was quantified as described previously [57]. Release of fluorescent 7-amino-4-methylcoumarin (AMC) after cleavage from Ac-DEVD-AMC was quantified over 4 h. Apoptosis was also quantified by flow cytometry with Annexin V and PI. For staining 1 × 10^5^ cells were incubated with 80 μg/ml PI and 9.6 μg/ml Annexin V conjugated to PacificBlue (No. 640917, Biolegend, San Diego, CA, USA) in Annexin V binding buffer (No. 422201, Biolegend) for 15 min at room temperature. Cells were analysed on a FACS Canto II (BD Biosciences, Franklin Lakes, NJ, USA). 10000 cells per sample were analysed. Data analysis was performed using FCS Express 4 Flow software (De Novo Software, Los Angeles, CA, USA).

### Cell cycle analysis

For cell cycle analysis 4 × 10^5^ cells treated as indicated were lysed in 400 μl hypotonic citrate buffer (0.1% sodium citrate, 0.1% Triton X-100) containing 50 μg/ml PI for 30–60 min. Following staining, intact nuclei (as discriminated by PE-area versus PE-width gating) were directly analysed for DNA content by FACS. 30000 (HCT116) or 50000 (MDA-MB-231) nuclei per sample were analysed.

### Western blotting and immunoprecipitation

Whole cell extracts were prepared in RIPA lysis buffer and processed for Western blotting essentially as described previously [57]. Monoclonal antibodies used for p53 and MDM2 were from Millipore (Billerica, MA, USA) or Santa Cruz (Dallas, TX, USA), respectively. All other primary antibodies were polyclonal. Detailed antibody information is available from the authors upon request. For co-IP, 300 μg of whole cell lysates were incubated with 0.5 μg p53 antibody. Control IPs were performed with the same amount of mouse IgG2a isotype control.

### Cell proliferation

To assess cell proliferation 2 × 10^5^ HCT116 or 3 × 10^5^ MDA-MB-231 cells were seeded in P60 cell culture dishes. To study effects of chemotherapy on proliferation, 1 × 10^5^ cells were reseeded in P60 cell culture dishes following incubation with etoposide or 5-fluorouracil on day 5 for 4 h. To assess the influence of irradiation on proliferation, 1 × 10^5^ cells were reseeded in P60 cell culture dishes 24 h after irradiation with 3 Gy on day 4. When control samples were confluent, cells were stained with trypan blue and viable cells counted using a Cellometer™ Auto T4 (Peqlab).

### Clonogenic survival assay

Depending on the experiment planned, 100 or up to 6400 cells in exponential growth were seeded in collagen precoated 6 well dishes. ERp57 knockdown was induced and cells were incubated for 16 h. The cells were irradiated with 1 or 3 Gy and incubated for 10 d. Cells were fixed with 0.25% paraformaldehyde and 70% ethanol and stained with Coomassie. Colonies of more than 50 cells were counted manually. Survival was calculated as number of colonies divided by cells seeded x plating efficiency as described previously [58].

### Immunofluorescence

To study the subcellular localization of ERp57, 1 × 10^5^ HCT116 cells were seeded onto glass cover slides coated with rat collagen-I (Biozol, Eching, Germany). pEGFP-N1-ERp57 encoding ERp57 with a C-terminal GFP was generated by PCR cloning. Following transfection cells were treated as indicated, incubated with 1 μM ER-Tracker™ Blue-White DPX (Invitrogen) and 5 μM DRAQ5™ (Thermo Scientific), fixed with 4% paraformaldehyde and analysed using a Zeiss LSM510 confocal microscope.

### Cellular fractionation

To separate the cytoplasm from other cellular compartments HCT116 cells were suspended in hypertonic buffer (5 mM sucrose, 1 mM HEPES pH 7.4) containing 50 μg/ml digitonin for 90 sec. The cytoplasmic fraction was collected following centrifugation with 800 × g for 1 min. Residual compartments were lysed in RIPA.

### XBP1 splicing

Total RNA from HCT116 cells was prepared using RNeasy Mini Kit (Qiagen, Hilden, Germany). Reverse transcription was performed using the iScript™ Select cDNA Synthesis Kit (Bio-Rad, Hercules, CA, USA). For detection of spliced and unspliced XBP1 cDNA PCR was performed using forward primer 5′-AAACAGAGTAGCAGCTCAGACTGC-3′ and reverse primer 5′-CCTTCTGGGTAGACCTCTGGGAG-3′. The PCR products of XBP1 and β-actin were visualized on 2.5% agarose gels.

### Reporter gene assays

5 × 10^4^ cells were seeded into 24-well plates. 24 h after knockdown induction, the cells were either co-transfected with 500 ng of the reporter plasmid p5xATF6-GL3 (#11976, Addgene) and 100 ng pGL4.74 (expressing renilla luciferase) or transfected with pFLAG-XBP1u-FLuc (#31239, Addgene). 16–24 h after transfection cells were treated as indicated. Firefly and renilla luciferase activities were quantified using a Dual-Luciferase Reporter Assay (Promega) on a TD 20/20 luminometer (Turner Designs, Sunnyvale, CA, USA).

### Statistical analysis

All experimental results were confirmed in at least two independent experiments unless otherwise indicated. Data presented in bar graphs include at least three independent samples. Bars represent mean plus standard deviation. Comparison of two groups was performed by application of Student's *t*-test using GraphPad Prism 5 (GraphPad Software, San Diego, CA, USA). Data were expressed as the mean + s.d. In all figures * indicates *p* < 0,05, while ** indicates a *p*-value of < 0,01 and ***p < 0,001.
